# Blood–Brain Barrier Permeability in ESKD—A Proof-of-Concept Study

**DOI:** 10.1681/ASN.0000000000000167

**Published:** 2023-07-04

**Authors:** Aditi Gupta, Aanya Bansal, Kate Young, Archana Gautam, Joseph Donald, Branden Comfort, Robert Montgomery

**Affiliations:** 1 Division of Nephrology and Hypertension and the Jared Grantham Kidney Institute, University of Kansas Medical Center, Kansas City, Kansas; 2 Department of Internal Medicine, University of Kansas Alzheimer's Disease Research Center, Kansas City, Kansas; 3 Department of Neurology, University of Kansas Alzheimer's Disease Research Center, Kansas City, Kansas; 4 Department of Biostatistics and Data Science, University of Kansas Medical Center, Kansas City, Kansas; 5 Department of Radiology, University of Kansas Medical Center, Kansas City, Kansas

**Keywords:** chronic dialysis, cognition, dementia

The blood–brain barrier (BBB) is a highly selective mechanical and physiological structure that regulates the passage of solutes between the blood and the brain. Increased BBB permeability is an early marker of cognitive decline and dementia^1^ and is implicated in cognitive impairment in kidney disease.^2^ BBB permeability is traditionally measured with dynamic contrast enhanced (DCE) magnetic resonance imaging (MRI).^3^ Gadolinium-based contrast agents, however, can be harmful in ESKD^4,5^ and can result in gadolinium accumulation in the brain,^6^ especially in individuals with increased BBB permeability. 99m technetium (Tc) diethylenetriaminepentaacetic acid (DTPA) single photon emission computed tomography (SPECT) can also measure BBB, without the need for gadolinium-based contrast agent. SPECT uses the same fundamental principles and chelating agent DTPA as DCE MRI but uses 99m Tc instead of gadolinium. Like gadolinium, 99m Tc does not cross an intact BBB, and appearance of 99m Tc in the brain indicates increased BBB permeability.

In this novel, cross sectional, proof-of-concept, single-center study, we for the first time, measured BBB permeability in ESKD. We measured 99m Tc DTPA SPECT derived mean standardized uptake values (SUVs) in ten ESKD and ten control adult participants between the ages of 18–65 years. Control participants required an estimated glomerular filtration rate >60 ml/min. Exclusion criteria included weight >500 lbs, known brain lesion or traumatic brain injury, or currently pregnant or breastfeeding state. All participants underwent a brain SPECT imaging. In addition, all participants underwent the National Alzheimer's Coordinating Center Uniform Data Set telephone cognitive battery (T-cog).^7^ For patients on in-center hemodialysis, T-cog was administered on nondialysis days.

Table 1 compares the demographics and clinical characteristics of the two groups. Patients with ESKD had a higher SUV (0.27±0.06) compared with the controls (0.17±0.04) (Figure 1A). Figure 1B shows the SUVs in relation to the age of the participants. While increased BBB is usually seen in older individuals in the general population, even younger patients with ESKD had a high BBB permeability. The difference BBB permeability is not appreciated visually and has to be calculated with a software. Although, ESKD overall performed slightly worse than the control group in many categories, T-cog scores were not statistically different between the two groups (Table 1). There was a high correlation between immediate recall (*r*=−0.806) and delayed recall (*r*=−0.731) and SUV and a moderate correlation between digit span forward (*r*=0.636), digit span backward (*r*=0.645), verbal fluency (*r*=0.506), and verbal naming (*r*=0.443) and SUV.

**Table 1.** Demographic and clinical characteristics of study participants

Participant Characteristic	ESKD (*n*=10)	Control (*n*=10)	*P* Value
Age (yr), mean (SD)	49.1 (12.0)	52.3 (12.2)	0.65^a^
BMI, mean (SD)	31.5 (7.0)	31.1 (9.3)	0.91^b^
Sex, male, *n* (%)	6 (60)	3 (30)	0.37^c^
Cause of ESKD, *n* (%)			
Diabetes	6 (60)		
ADPKD	2 (20)		
Others	2 (20)		
Current dialysis access, *n* (%)			
Catheter	6 (60)		
AVF	3 (30)		
AVG	1 (10)		
Mode of dialysis, *n* (%)			
In-home HD	1 (10)		
In-center HD	4 (40)		
PD	5 (50)		
Time on dialysis (yr), mean (min-max)	2.1 (0–7)		
Heart attack, *n* (%)	2 (20)	0 (0)	0.47^c^
Psychiatric disorder, *n* (%)	5 (50)	6 (60)	1.00^c^
Depression, *n* (%)	3 (30)	6 (60)	0.37^c^
SUVs, mean (SD)	0.27 (0.06)	0.17 (0.04)	<0.001^b^
T-cog tests, *n* (%)			
MoCA blind	20.0 (2.7)	20.2 (1.5)	0.67
Immediate recall	22.2 (7.1)	25.8 (6.3)	0.16
Delayed recall	19.8 (6.8)	22.7 (7.6)	0.24
Digit span forward	9.6 (2.5)	8.0 (2.4)	0.21
Digit span backward	7.5 (2.1)	8.2 (2.5)	0.42
Verbal fluency	11.0 (4.7)	14.5 (5.4)	0.29
Oral trailmaking A^d^	6.9 (1.9)	6.9 (1.7)	1.00
Oral trailmaking B^d^	33.9 (18.1)	27.4 (13.3)	0.31
Category fluency animals	22.1 (8.3)	26.0 (4.6)	0.27
Category fluency vegetables	13.3 (4.2)	15.9 (3.6)	0.13
Verbal naming	48.3 (2.4)	48.3 (2.0)	0.87

BMI, Body mass index; ADPKD, autosomal dominant polycystic kidney disease; AVF, arteriovenous fistula; AVG, arteriovenous graft; HD, hemodialysis; PD, peritoneal dialysis; SUV, standardized uptake value; MoCA, Montreal cognitive assessment.

a Wilcoxon rank-sum test.

b Wilcoxon rank-sum exact test.

c Fisher exact test.

d Higher scores indicate longer time needed to complete the test and hence, worse cognitive function. For all other cognitive tests, higher scores indicate better cognitive function.

**Figure 1.**
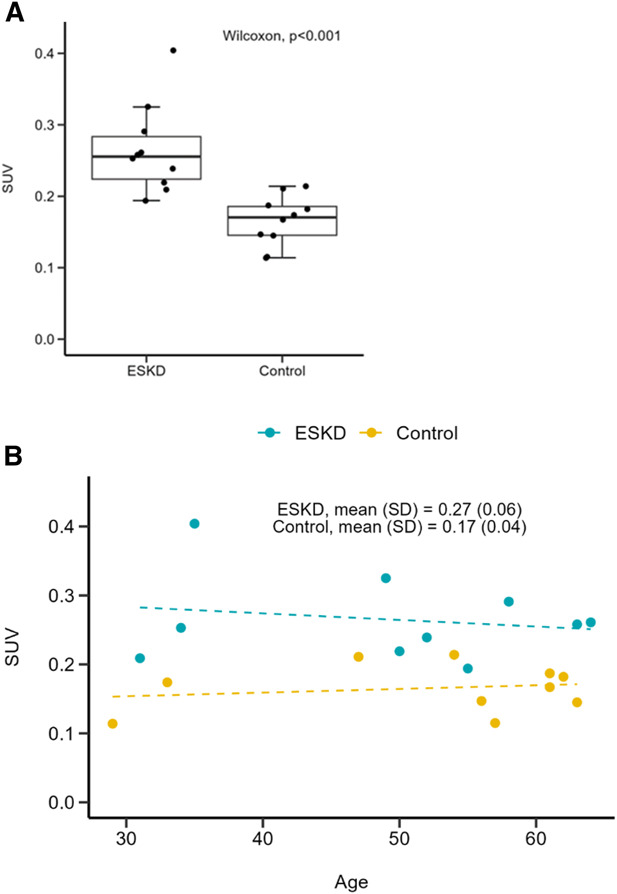
**Scatterplots displaying individual participants' mean SUV in the ESKD and control groups.** (A) Box and whisker plots for SUV in ESKD and control groups. (B) SUV as a function of age and group (controls: yellow circles, ESKD: blue circles). The dashed lines represent the estimated group means across age. SUV, standardized uptake value.

In addition, we used our global hypothesis test^8^ to determine whether our hypotheses (higher SUV and worse T-cog scores in ESKD compared with controls) were borne out in the data. The global hypothesis test does not establish causality, it simply determines whether data in the pilot study indicate whether the theory is worthy of a future larger study. Of the 12 end points in the prediction test, the difference between groups were correctly predicted in nine, indicating the data support our hypotheses (*P* = 0.048). Adjusting for age resulted in stronger results, with 11 of 12 end points being correctly predicted (*P* = 0.003).

The significance of this proof-of-concept study is two-fold: (*1*) It indicates that BBB permeability may be increased in ESKD where it may mediate cognitive impairment, and (*2*) it tests a novel protocol to assess BBB permeability, especially in individuals where DCE MRI may not be suitable. We have previously shown alterations in cerebral blood flow, white matter integrity, and brain neurochemicals in ESKD that suggest disruption of the BBB in ESKD.^9^ This study, for the first time, shows increased BBB permeability in ESKD and supports our previous findings. Our data are also consistent with previous findings on cognitive function in ESKD. Memory is affected in ESKD,^10^ and we observed a high negative correlation between SUV and immediate and delayed recall (neuropsychological tests for memory).

Cognitive impairment is highly prevalent in ESKD. Increased BBB is an early biomarker of future dementia and perhaps the underlying mechanism for age-related decline in cognitive function. BBB dysfunction and increased permeability allows harmful substances, including uremic metabolites in the systemic circulation, to enter the brain and invoke neuronal and synaptic injury and dysfunction. Because uremic metabolites are known to be neurotoxic and contribute to uremic encephalopathy, increased BBB permeability may be more important in kidney disease than in the general population. Increased BBB permeability and its association have been shown in animal models of ESKD.^2^ This is the first human study showing increased BBB in humans with ESKD. This study is an initial step in assessing BBB permeability as a prognostic biomarker to assess the risk of cognitive impairment and to monitor response to intervention to prevent or slow cognitive decline in ESKD. Cognitive changes take time, and subtle cognitive changes can be hard to detect. BBB permeability may be a preclinical predictor for future cognitive decline that could allow early intervention to prevent future dementia.

Previous studies have used SPECT in stroke^11^ and in assessing the association of poststroke seizures with BBB permeability^12^ by comparing the affected hemisphere with contralateral hemisphere. However, this is the first study quantifying BBB permeability in the whole brain. Replication of the age-associated increase in BBB permeability in our study increases the confidence for measurement of BBB permeability using SPECT imaging.

Being a pilot study, it is limited by the small sample size and heterogeneity of participants. Cognitive impairment in ESKD is complex and multifactorial. Future studies are needed to confirm these associations and assess the role other factors such as systemic inflammation, cerebral blood flow, and its changes with dialysis, comorbidities, and stroke, which was beyond the scope of this study. This study builds on our current understanding of cognitive impairment in ESKD and highlights a potential underlying mechanism for cognitive impairment in ESKD.

## Methods

Patients were enrolled through our hospital dialysis units and through institutional review board–approved study fliers posted within the university campus and from the primary care clinics of a large academic center. The study was approved by the Institutional Review Board of the University of Kansas Medical Center (STUDY0014699 and approved on March 18, 2021), and all participants signed an informed consent.

All participants underwent SPECT brain imaging on a GE NM/CT 870 DR CZT SPECT/CT scanner. 20 mCi of 99m Tc DTPA was injected intravenously and flushed with 10 ml saline. Attenuation-corrected computed tomography images were collected for identification and segmentation of bone from brain tissue with the following parameters (matrix=128×128, 15 seconds per stop, 120 total frames, 3° acquisition). DTPA images were collected 30 minutes postinjection using a low-energy high resolution and sensitivity collimator with the following parameters (slice thickness=3.75 mm, scan type=helical, x-ray voltage=120 kV, x-ray current=20 mA, matrix=128×28, reconstruction matrix=512×512, center of rotation correction, 15 seconds per stop, 120 total frames, 3° acquisition, window=20%). DTPA images were then segmented using the computed tomography bone and brain filters, and mean SUVs were calculated for the brain segment within the GE XELERIS software suite.

We also conducted phone-based cognitive function assessments (T-cog), the National Alzheimer's Coordinating Center Uniform Data Set telephone cognitive battery. T-cog included Montreal cognitive assessment (MoCA) blind, immediate recall, delayed recall, digit span forward, digit span backward, verbal Fluency, oral trailmaking A, oral trailmaking B, category fluency for animals, category fluency for vegetables, and verbal naming. Supplemental Table 1 describes these tests and their scoring. For example, MoCA blind is a modified version of the original MoCA test adapted for telephone administration. It has a maximum score of 22 and a cutoff score of 18 or more for normal cognition. T-cog was performed on a separate phone call scheduled after the SPECT. The study coordinator who performed T-cog was trained by neuropsychologists and has more than 10 years of experience performing neurophysiological tests for clinical trials in cognitive impairment. To avoid any distractions or interruptions during the T-cog, the coordinator performed the T-cog when both the coordinator and the participant were in a private room.

## Statistical Analysis

We performed descriptive analyses for demographic and clinical variables and their comparison between patients with ESKD and controls with ESKD. Our primary aim was the difference in mean SUV between groups. We used the Wilcoxon rank-sum test to assess differences in SUV and T-cog scores in the two groups. We also assessed correlation between mean SUV in the ESKD group and the T-cog scores. In addition, we used our global hypothesis test to determine whether our hypotheses (higher SUV and worse T-cog scores in ESKD compared with controls) were borne out in the data. The null hypothesis for the global hypothesis test was that the study hypotheses provide no better predictions of end points (SUV and T-cog scores in the two groups) than chance. Rejecting the null hypothesis would provide initial evidence that our research hypotheses are supported by the data. These analyses were intended to provide more information to plan a future study. *P* values are provided to help understand the magnitude of the effects and should be interpreted in light of the small sample size and lack of multiplicity correction.

## Supplementary Material

## Data Availability

The data presented in this study are available on request from the corresponding author. The data are not publicly available due to privacy or ethical restrictions.

## Supplemental Material

Supplemental Table 1. Description of neuropsychological tests used in the cognitive assessments (T-cog).
