# Supplementary material for: Blood–Brain Barrier Permeability in ESKD—A Proof-of-Concept Study
Source: J Am Soc Nephrol. 2023 Jul 4;34(9):1508–11. doi: 10.1681/ASN.0000000000000167 (PMC10482059; doi:10.1681/ASN.0000000000000167)
Supplement: Supplementary file 1 [file jasn-34-1508-s001.pdf]

Supplementary Table 1: Description of neuropsychological tests used in the cognitive assessments (T-cog)

Test	Administration time	Description	Scoring
MoCA blind	10 minutes	Adapted version of the original 30-item MoCA for use over the telephone or for individuals with poor vision.	Higher scores indicate better cognitive function. Maximum score 22, a score of 18 or more is considered normal.
Immediate recall	5 minutes (with 20-minute delay)	Assesses the ability to recall a short story. The examiner reads the story to the participant. Immediately after, the participant is asked to retell the story from memory. After a 20-minute delay, the participant is asked to repeat the story.	Higher scores indicate better cognitive function. Maximum score 44.
Delayed recall	2 minutes	Administered 20 minutes after the immediate recall. The participant recalls the story that was read to the participant at the beginning of the testing session, during the immediate recall.	Higher scores indicate better cognitive function. Maximum score 44.
Digit span forward and backward	5 minutes	Numbers for forward and backward span tests are presented, with sequences in ascending order of difficulty. Two trials are administered at each sequence length. The longest sequence repeated correctly is used for scoring.	Higher scores indicate better cognitive function.
Verbal Fluency	5 minutes	The participant names items that begin with a certain letter of the alphabet. The number of unique responses named is scored.	Higher scores indicate better cognitive function.
Oral Trailmaking A and B	5 minutes	Brief measure assessing mental sequencing and switching. The time (seconds) needed to complete the given number/letter series is recorded.	Higher scores indicate worse cognitive function. Maximum score 100 seconds for Trailmaking A, Maximum score 300 seconds for Trailmaking B.
Category Fluency	5 minutes	The participant is asked to name various items of a given semantic category (animals or vegetables), and the number of unique responses named is scored.	Higher scores indicate better cognitive function. Maximum score 77.
Verbal Naming	10 minutes	An auditory naming test where participants name a specific item after hearing a description	Higher scores indicate better cognitive function. Maximum score 50.

Obtained from the NACC website
